# Environmental Remediation of the difficult-to-return zone in Tomioka Town, Fukushima Prefecture

**DOI:** 10.1038/s41598-020-66726-y

**Published:** 2020-06-23

**Authors:** Limeng Cui, Yasuyuki Taira, Masahiko Matsuo, Makiko Orita, Yumiko Yamada, Noboru Takamura

**Affiliations:** Department of Global Health, Medicine and Welfare, Atomic Bomb Disease Institute, Nagasaki University Graduate School of Biomedical Sciences, 1-12-4 Sakamoto, Nagasaki City, Nagasaki Prefecture, 852-8523 Japan

**Keywords:** Environmental sciences, Risk factors, Physics

## Abstract

Temporal variations in ambient dose rates in a restricted area designated as “difficult-to-return” for residents of Tomioka Town, Fukushima Prefecture were evaluated in a car-borne survey during 2018–2019. The median dose rates in the “Decontaminated area” in the difficult-to-return zone decreased rapidly from 1.0 μSv/h to 0.32 μSv/h; however, the median dose rates in the “Non-decontaminated area” and “Radioactive waste storage area” fluctuated between 1.1–1.4 μSv/h and 0.46–0.61 μSv/h, respectively. The detected rate of the cesium-137 (^137^Cs) (^137^Cs-detected points per all measuring points) in the “Decontaminated area” also decreased rapidly from 64% to 6.7%, accompany with decreasing in ambient dose rates. On the other hand, the detection of ^137^Cs in the “Radioactive waste storage area” and “Non-decontaminated area” decreased from 53% to 17% and 93% to 88%, respectively. We confirmed that the dose rates in the Decontaminated area dramatically decreased due to decontamination work aiming to help residents return home. Moreover, the estimated external exposure dose of workers during the present survey was 0.66 mSv/y in the Decontaminated area and 0.55 mSv/y in the Radioactive waste storage area, respectively. This case of Tomioka Town within the “difficult-to-return zone” may be the first reconstruction model for evaluating environmental contamination and radiation exposure dose rates due to artificial radionuclides derived from the nuclear disaster.

## Introduction

The Great East Japan Earthquake (magnitude 9.0) and subsequent tsunami on March 11, 2011 caused an accident at the Fukushima Daiichi Nuclear Power Station (FDNPS) that resulted in various radionuclides including cesium-134 (^134^Cs), cesium-137 (^137^Cs) and iodine-131(^131^I) being released into the atmosphere and eventually depositing on land and at sea in the surrounding areas^1^. The United Nations Scientific Committee on the Effects of Atomic Radiation estimated the total release of ^134^Cs (half-life: 2.1 y), ^137^Cs (half-life: 30 y) and ^131^I (half-life: 8 d) to be 9.0, 8.8 and 120.0 petabecquerels (PBq), respectively^1^. The Japanese government, municipalities and private companies have carried out environmental and individual radiation monitoring programs to confirm the radiation levels in the affected areas^2,3^. More than 8 years have passed since the accident and it has been confirmed that artificial radionuclides with a relatively long half-life such as ^134^Cs and ^137^Cs still exist in soil and plant samples collected around the FDNPS^1–3^.

Environmental monitoring in Fukushima Prefecture have been carried out by many methods (the airborne survey by monitor stations and personnel, vehicle-borne survey, aerial-vehicle survey and radionuclide analysis of the environmental samples such as soils, sediments and foodstuffs)^3–9^. These surveys and the collected data are extremely important to precise evaluation of environmental remediation in the affected areas. Following the FDNPS accident, residential areas, farmlands, forests close to residential areas, and roads within the evacuation order areas were extensively decontaminated by March 19, 2018. This excluded an area designated as “difficult-to-return” for residents, an area in which entry and lodging are basically still prohibited^10^. According to the *Act on Special Measures for the Reconstruction and Revitalization of Fukushima* outlined in 2017, six municipalities, including Tomioka Town, are making plans to construct a Special Reconstruction and Revitalization Base aiming to lift evacuation orders and allow the residents to return to home^10^.

**Figure 1:**
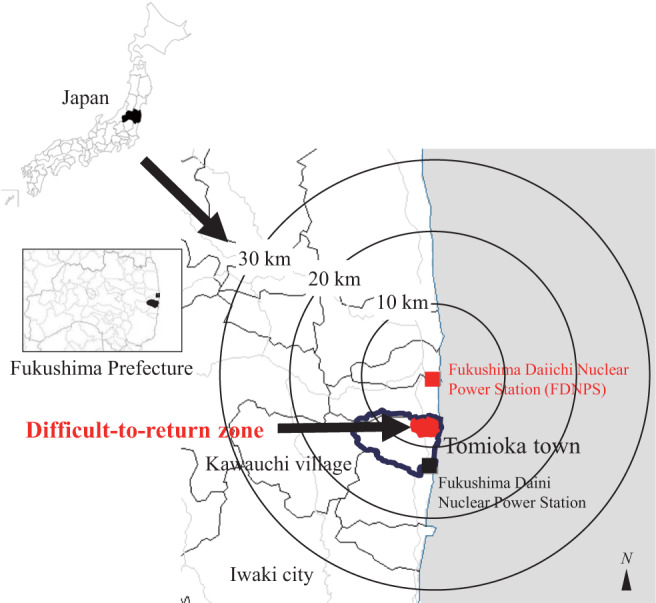
Location of Tomioka Town, Fukushima Prefecture, Japan. The second author (Y.T.) created the map using GIS software (Green Map III, Tokyo Syoseki, Tokyo, Japan. https://shop.tokyo-shoseki.co.jp/map). Reprinted from Green Map III under a CC BY license, with permission from Tokyo Shoseki Co., Ltd.; original copyright 2003.

The National government established areas estimated more than 50 mSv/y in the annual cumulative dose, as of March 2012 as the difficult-to-return zone. Tomioka Town was rearranged into a residential zone and the difficult-to-return zone depending on contrasted levels of the annual cumulative dose (Fig. 1). The difficult-to-return zone is about 8.5 km^2^ and about 4,800 people were living there before the disaster^11^.

Long-term environmental monitoring as well as further decontamination efforts should continue around the FDNPS, including Tomioka Town. On the other hand, the external exposure level and the decontamination effects on landscape within the difficult-to-return zone have not been evaluated concretely, although data from the literature, databases and websites have been reported by the national and local governments^4–9,12,13^. Especially, recent reports on the decontamination effect on landscape are not sufficiently published^14^.

Therefore, in the present study, we carried out a detailed and high-frequency radiation monitoring program using a car-borne survey to provide relatively high-density data. We also evaluated the effects of decontamination efforts, such as reductions in ambient and radiocesium dose rates, in three areas (“Decontaminated area”, “Radioactive waste storage area” and “Non-decontaminated area”) with markedly different characteristics in the difficult-to-return zone in Tomioka Town.

## Results

### Ambient dose rates

**Figure 2:**
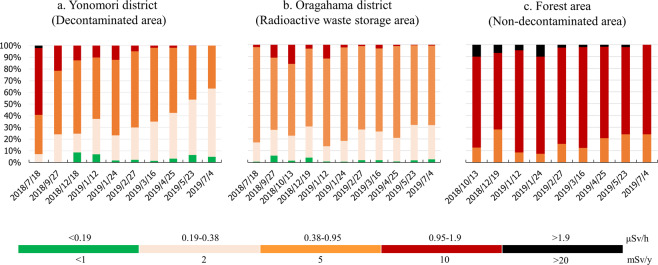
Relative frequencies of ambient dose rates in the Difficult-to-return zone in Tomioka Town, Fukushima Prefecture from July 2018 to July 2019. (**a**) Yonomori District (Decontaminated area); (**b**) Oragahama District (Radioactive waste storage area); (**c**) Forest area (Non-decontaminated area).

The frequency distributions of the ambient dose rates within the difficult-to-return zone of Tomioka town were illustrated in Fig. 2. In the decontaminated area, the proportion of the locations with dose rates more than 0.95 μSv/h largely dropped from 59.2% in July 2018 to 0% in July 2019. The dose rates mainly concentrated range from 0.38 to 0.95 μSv/h (61%-81%) in the radioactive waste storage area. In the non-decontaminated area, from 72% to 93% measurement points were higher than 0.95 μSv/h during the research period.

Relatively higher dose rates were observed in the Non-decontaminated area with median dose rates ranging from 1.1 to 1.4 μSv/h during the research period (Table 1). Likewise, the median dose rates ranged from 0.46 to 0.61 μSv/h in the Radioactive waste storage area. On the other hand, the ambient dose rates of the Decontaminated area dramatically decreased from 1.0 μSv/h in July 2018 to 0.32 μSv/h in July 2019. On the basis of the slope of the regression line, ambient dose rates in the last surveys decreased to 28.1%, 78.9% and 72.1% of those in the first surveys in the Decontaminated area, Radioactive waste storage area and Non-decontaminated area, respectively (Table 1).

**Table 1:** Ambient dose rates in the three districts of the Difficult-to-return zone in Tomioka Town from July 2018 to July 2019.

	Yonomori district (Decontaminated area)			Oragahama district (Radioactive waste storage area)			Forest area (Non-decontaminated area)		
	Points^a^	Median(min-max) (μSv/h)	Decreasing proportion (%)	Points	Median(min-max) (μSv/h)	Decreasing proportion (%)	Points	Median(min-max) (μSv/h)	Decreasing proportion (%)
2018/7/18	922	1.0 (0.24–2.82)	100	592	0.54 (0.15–1.1)	100	N/A^b^	N/A	N/A
2018/9/27	748	0.70 (0.19–1.3)	56.7	622	0.59 (0.11–1.1)	91.1	N/A	N/A	N/A
2018/10/13	N/A	N/A	N/A	510	0.55 (0.13–1.8)	109.5	159	1.4 (0.43–2.4)	100
2018/12/19	1034	0.57 (0.13–1.3)	49.6	638	0.54 (0.12–1.9)	91.5	157	1.2 (0.39–2.4)	80.4
2019/1/12	1408	0.53 (0.14–1.5)	44.6	744	0.61 (0.17–1.7)	106.6	189	1.4 (0.53–2.7)	92.8
2019/1/24	942	0.62 (0.15–1.5)	49.6	600	0.57 (0.15–1.2)	95.3	148	1.4 (0.52–2.6)	93.3
2019/2/27	826	0.51 (0.13–1.4)	42.8	525	0.50 (0.14–1.0)	85.5	127	1.3 (0.40–2.2)	85.4
2019/3/16	1508	0.46 (0.14–1.5)	37.7	849	0.51 (0.12–1.3)	85.4	145	1.3 (0.43–2.2)	79.4
2019/4/25	1187	0.41 (0.12–1.5)	39.2	725	0.52 (0.14–1.2)	84.0	121	1.3 (0.44–2.0)	79.7
2019/5/23	1102	0.36 (0.12–1.1)	35.0	597	0.46 (0.16–1.0)	76.9	155	1.1 (0.38–2.1)	73.1
2019/7/4	1188	0.32 (0.12–0.94)	28.1	586	0.46 (0.13–1.2)	78.9	138	1.1 (0.39–1.8)	72.1

^a^measurement points. ^b^unable to survey.

Ambient dose rates were significantly higher in the Non-decontaminated area than in the other two areas (*p* < 0.001). In the surveys during 2018 and on January 24, 2019, the dose rates in the Decontaminated area were significantly higher than those in Radioactive waste storage area (*p* < 0.001). However, in the survey on January 12, 2019 and the four surveys after March 2019, the statistical results indicated the dose rates in the Decontaminated area fell below those of the Radioactive waste storage area (*p* < 0.001).

**Figure 3:**
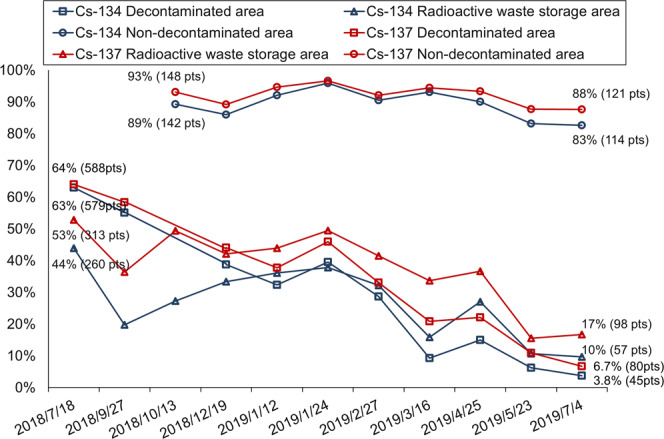
Proportion of localities where radiocesium could be detected in the Difficult-to-return zone in Tomioka Town from July 2018 to July 2019. Percentage is shown to the rate of detected points.

Furthermore, we analyzed the spectrum of the ambient gamma-ray flux (mainly artificial radionuclides such as radiocesium) using the Radi-probe system. The proportion of measurement points where radionuclides could be detected compared to all measurement points is shown in Fig. 3. The number of be detected points of ^137^Cs ranged from 64% (588 in 922 points) to 6.7% (80 in 1188 points), 53% (313 in 592 points) to 17% (98 in 586 points) and 93% (148 in 159 points) to 88% (121 in 138 points) in the Decontaminated area, Radioactive waste storage area, and Non-decontaminated area, respectively, and those of ^134^Cs ranged from 63% (579 in 922 points) to 3.8% (45 in 1188 points), 44% (260 in 592 points) to 10% (57 in 586 points), 89% (142 in 159 points) to 83% (114 in 138 points), respectively (Fig. 3). In the present study, radiocesium fallout driven from the FDNPS accident was clearly detected by this car-borne survey system as one source of the ambient dose rate, even after 8 years from the Accident.

### External effective doses

We calculated the annual external effective doses of decontamination workers and estimated that the median doses from July 2018 to July 2019 were 0.66 mSv/y for those working in the Decontaminated area and 0.55 mSv/y in the Radioactive waste storage area, respectively. Also, for residents who are going to return to the Decontaminated area, on the basis of the ambient rates in July 2019, we estimated that the median external effective dose of indoor workers was 0.69 mSv/y and that of outdoor workers was 0.87 mSv/y, respectively.

## Discussion

In the present study, the median dose rate in the whole difficult-to-return zone was 0.46 µSv/h in July 2019, which showed a clear decrease. One car-borne survey in the difficult-to-return zone of Namie Town near Tomioka Town (within the 20 km of the FDNPS) reported absorbed dose rates ranging from 1 to 5 μGy/h in 2017^15^. Our previous handheld measurements showed the median dose rate was 2.3 µSv/h in the difficult-to-return zone of Tomioka Town in 2017^16^.

The dose rates in the Decontaminated area decreased faster than those in the Radioactive waste storage area and Non-decontaminated area from July 2018 to July 2019. Significant differences in ambient dose rates were observed among surveys in the Decontaminated area, Radioactive waste storage area and Non-decontaminated area (*p* < 0.001). Noticeable fluctuations in dose rates in the Radioactive waste storage area and Non-decontaminated area were observed. Also, a relatively stable downward trend was observed in the Decontaminated area.

The main reason for the decrease in dose rates over this 1-year period in Yonomori District is the decontamination efforts which have focused on removing deposits from roofs, decks and gutters; wiping off roofs and walls; high-pressure washing of houses and buildings; mowing lawns; removing fallen leaves and stripping topsoil in gardens; removing deposits in ditches and high-pressure washing of roads^10,17,18^ (Supplementary Fig. S1). In our previous report, the effectiveness of removing topsoil for decontamination, and the positive relationship between soil radioactivity and air dose rates have been reported previously^16^. One report suggested that the total ^137^Cs content in soils was 1200 Bq/kg on average (value range: 20–4400 Bq/kg), which was an 80% decrease from the values determined before the decontamination within agricultural fields in Tomioka Town^19^. The Ministry of the Environment, Japan reported that due to decontamination, the ambient rate 1 m above the ground surface was reduced by 60% in residential areas, and 42% on the roads^20^. Another report suggested that the average dose rate in the Decontaminated area was about 20% lower than that in the Non-decontaminated area^21^. Our study also showed that the dose decreased by 71.9% within 1 year of decontamination efforts in areas where the initial dose rate was 1.0 μSv/h (median) in the Decontaminated area (Yonomori District). In the present study, the small range and high frequency of sampling points with the Radi-probe system could concretely estimate the effects of decontamination.

Moreover, the physical decay of the ambient dose rates was calculated using dose conversion coefficients under the assumption that the depth profile of radiocesium did not change with time and the initial radioactivity of ^134^Cs and ^137^Cs were 9.0 and 8.8 PBq, respectively^1,22^. In the present study, the physical decay of radiocesium was estimated to be 7.5% from July 2018 to July 2019 (Supplementary Table S1). The reduction rates during research period in the Radioactive waste storage area and Non-decontaminated area were 21.1% and 27.9%, respectively. Our results showed that the reduction rates of radiocesium in all three districts were noticeably faster than its physical decay.

In Yonomori District, the decreasing time trends of the confidence levels of radiocesium were consistent with the decreasing time trends of the ambient dose rates. Furthermore, the distribution of ^137^Cs in the Non-decontaminated area remained at a high level (Fig. 3). The confidence level of ^137^Cs in the Non-decontaminated area, which is mainly covered by forest, showed a relatively slower decreasing trend compared with other areas. Previous studies also reported a longer ecological half-life in forested areas and suggested that the accumulation of radiocesium in association with the self-decontamination processes of forest canopies affects the temporal evolution of the ambient dose rate at the forest floor^23–26^.

Previous studies indicated that the dose rates decreased due to radioactive decay, natural weathering effects, penetration of radiocesium into the ground, land use and decontamination^15,27–29^. The forest ecosystem also retains radionuclides; decreases in dose rates are typically slower than those in urban areas and annual doses can be very high^23,30–32^. Some studies have suggested that the rate of the decrease in radiocesium doses in Fukushima was faster than that in the forests contaminated by the Chernobyl nuclear accident^33^. Furthermore, Kato *et al*. reported that the rate of the decrease in radiocesium doses in mixed broad-leaved forests and deciduous broad-leaved forests during 2011–2016 was approximately 20% higher than the physical decay rate of radiocesium, which corroborates our findings (20.4%)^24^.

In the Radioactive waste storage area, the ambient dose rates were sometimes higher in later surveys than in the first survey, which might result from radiocesium being resuspended in the air with dust particles due to dump truck traffic performing decontamination work and/or meteorological events^34,35^. The decreasing proportion of ambient dose rates in the Radioactive waste storage area suggested that human activities such as a contaminant waste storage project may lead to a 0–10% fluctuation in ambient dose rates (Table 1).

In the present study, the estimated annual effective dose of decontamination workers, as well as the residents of decontaminated areas, was lower than the annual effective dose limits recommended by the Japanese government^36^. Nevertheless, radiation safety education for workers is needed to appropriately protect them from radiation.

We could not carry out all of the car surveys on the same routes because the Decontaminated area was expanding with progression of the decontamination efforts and some roads were temporarily blocked during the decontamination work. Furthermore, the dose rate transition with the season and weather was difficult to identify through horizontal comparison over 11 surveys. However, the main artificial radionuclides, such as ^137^Cs, derived from the FDNPS accident could be analyzed to sufficiently low levels using the Radi-probe system. Moreover, the long-term follow-up monitoring in combination with various analytical apparatus and system such as car-borne survey and nuclides analysis of the environmental samples could be accurately evaluate the decontamination effects, external and internal radiation levels. These monitoring is extremely important for the reconstruction of affected areas around the FDNPS.

## Materials and Methods

### Survey location

**Figure 4:**
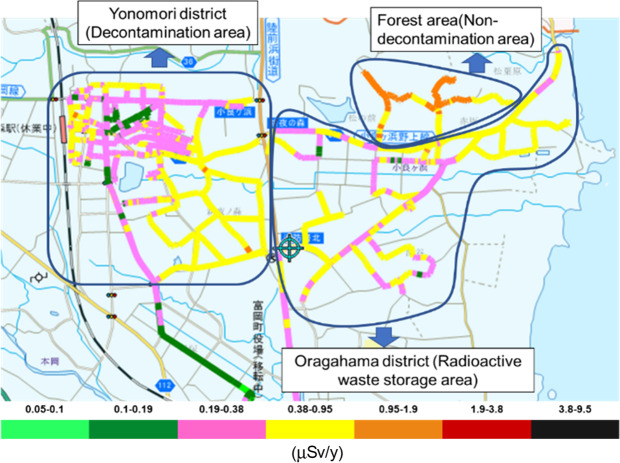
Real-time map of color-scaled ambient dose rates in the Difficult-to-return zone in Tomioka Town (May 2019). This map was modified by using PowerPoint software, from the map obtained by the car-borne survey using the Radi-probe system made in May 2019 (GIS software: Shobunsha Publications, Inc., Tokyo, Japan. https://www.mapple.co.jp/en/. The Radi-probe system: Chiyoda Technology Corp., Tokyo, Japan. http://www.c-technol.co.jp/eng). Blue lines show the three districts (Yonomori District: Decontaminated area; Oragahama District: Radioactive waste storage area, and Forested area: Non-decontaminated area). Reprinted from the map software for the Radi-probe system under a CC BY license, with permission (No. 61-G-081) from Shobunsha Publications, Inc., Tokyo, Japan; original copyright 2017 and Chiyoda Technology Corp., Tokyo, Japan.

The FDNPS (37°25′ N, 141°02’ E) is located on the east coast of Honshu Island, approximately 200 km northeast of Tokyo. Tomioka Town (public office: 37°20’ N, 141°0’ E) is located 8.5 km south of the FDNPS. In the present study, we measured ambient dose rates and artificial radionuclides (mainly radiocesium) derived from the FDNPS accident in the difficult-to-return zone of Tomioka Town from July 2018 to July 2019 (Fig. 4).

The different-to-return zone of Tomioka Town was divided by the main road between Yonomori District and Oragahama District, both of which are located within 10 km of the FDNPS (Fig. 3). Yonomori District was designated by the government as a reconstruction and revitalization area and main decontamination efforts started in July 2018^37^. The decontamination work involved cleaning paved surfaces and roadsides and street drains, topsoil removal, weeding and pruning trees, washing building surfaces and demolish building^17,38^. Part of Oragahama District was designated a radioactive waste storage area and was decontaminated in 2014; however, the forested area of this district has not been decontaminated since the accident. In the present study, Yonomori District is referred to as the Decontaminated area, the radioactive waste storage area in Oragahama District is referred to as the Radioactive waste storage area, and the forested area of Oragahama District is referred to as the Non-decontaminated area.

### Survey of ambient rates and radionuclides

We regularly measured the ambient dose rate from July 2018 to July 2019 (10 times in the Decontaminated area; 11 times in the Radioactive waste storage area; nine times in the Non-decontaminated area). The difficult-to-return zone of Tomioka Town was surveyed using a car-borne survey system, Radi-probe (Chiyoda Technology Corp., Tokyo, Japan. The handheld radiation detector model: HDS-101GN, Mirion Technologies, Inc., Japan)^6,39^. The Radi-probe system was installed in a vehicle and the meter’s detector was set on the front passenger seat about 1 m above the ground. The ambient dose rates were measured and position coordinates and a photo were automatically taken every 5 seconds in addition to spectrum segments every 0.2 seconds. Gamma detection was performed by a large Thallium doped Cesium Iodide scintillator with high sensitivity (Typical 1400 cps per µSv/h for ^137^Cs source). The measurable energy range of gamma-ray energy was 30 keV to 6 MeV, using a multichannel analyzer with 512 channels. Real-time maps with color-scaled ambient dose rates and gamma-ray energy spectra can be output. The detected energy peaks of radiocesium (^134^Cs and ^137^Cs) registered in the nuclear library (i.e., detected net count values) and their associated confidence intervals were obtained for the region of interest (with levels 1–10 used as reference values)^7,39^.

Generally, the car chassis and wall acted as a shield to radiation from outside. The shielding factors were estimated by taking measurements inside and outside the car in open and flat areas at a high of 1 m above the ground. Since many factors such as the type of car and the number of passengers could influence the shielding factors^40^, we calculated the shielding effects before each vehicle survey and the shielding factors were found to range from 1.1 to 1.6. For all surveys, vehicles were driven by the same person at a steady speed. The number of measurement points fluctuated due to restricted access to roads as decontamination efforts progressed. Combined with the output photos, the three districts were precisely divided. The measurement points ranged from 748 to 1408, 510 to 849 and 127 to 189 in the Decontaminated area, Radioactive waste storage area and Non-decontaminated area, respectively.

### Effective dose

Effective doses were determined for external exposure based on the following formula:

1 $${E}_{i}=({{\rm{D}}}_{{\rm{out}}}-{{\rm{D}}}_{{\rm{BG}}})\cdot {\rm{T}}\cdot {\rm{R}}$$

2 $${{\rm{E}}}_{{\rm{w}}}={\sum }_{i=1}^{12}{E}_{i}$$

3 $${\rm{E}}={{\rm{E}}}_{{\rm{out}}}+{{\rm{E}}}_{{\rm{in}}}$$

4 $${{\rm{E}}}_{{\rm{out}}/{\rm{in}}}=({{\rm{D}}}_{{\rm{out}}/{\rm{in}}}-{{\rm{D}}}_{{\rm{BG}}})\cdot {\rm{T}}\cdot {\rm{F}}\cdot {\rm{R}}$$

5 $${{\rm{D}}}_{{\rm{in}}}=r\cdot {{\rm{D}}}_{{\rm{out}}}$$

where ${E}_{i}$ is the estimated external effective dose (mSv/month by median); E_w_ is the external effective dose for decontamination workers (mSv/y); E is the external effective dose for residents who are going to return to the Decontaminated area (mSv/y); E_out/in_ is the external effective dose for outdoor and indoor workers; D_out/in_ is the dose rate for a height of 1 m above ground outside and inside the house (μSv/h); D_BG_ is 0.04 μSv/h, which was measured in the area of interest before the accident^41^; T is the work time, 240 d × 8 h (normal labor standards in Japan); F is the occupancy factor^1^; R is the age-dependent dose conversion coefficient for adults (0.6)^22,42^, and, *r* is the deposited gamma location factor for a wooden house (0.4)^43^.

### Statistical methods

All of the data were not normally distributed. The Mann-Whitney U and Kruskal-Wallis H tests were used to compare differences among the three areas in the same period and the time-trend within the same district. Regression lines were used to calculate the reduction rate of the average ambient dose rates.

## Supplementary information

Supplementary Information.
