# Supplementary material for: Environmental Remediation of the difficult-to-return zone in Tomioka Town, Fukushima Prefecture
Source: Sci Rep. 2020 Jun 23;10:10165. doi: 10.1038/s41598-020-66726-y (PMC7311413; doi:10.1038/s41598-020-66726-y)
Supplement: Supplementary file 1 — Supplementary Information. [file 41598_2020_66726_MOESM1_ESM.pdf]

Environmental Remediation of a Restricted Area in Tomioka Town, Fukushima Prefecture

Limeng Cui, Yasuyuki Taira*, Masahiko Matsuo, Makiko Orita, Yumiko Yamada and Noboru Takamura
Department of Global Health, Medicine and Welfare, Atomic Bomb Disease Institute, Nagasaki University, 1-12-4 Sakamoto, Nagasaki Prefecture 852-8523, Japan
*Corresponding. y-taira@nagasaki-u.ac.jp

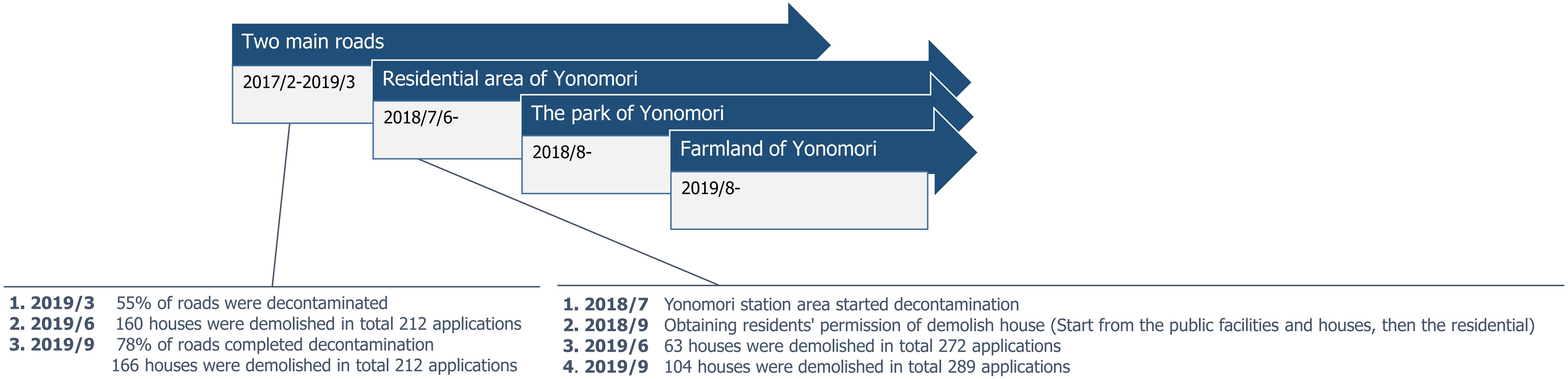

Supplementary information Figure 1; Decontamination schedule in Tomioka Town

An overview is shown as follow Figure 1[1-2].
1. Ministry of the Environment of Japan. Decontamination Guidelines. (2013). (Ref. 16 in the text)
2. Tomioka Town Office website (https://tomioka-radiation.jp/josen.html) (in Japanese)

Supplementary information Table 1; physical decay

Release date	survey date	Days after the release	Years after release	The radioactivty of Cs-134 (PBq)	The radioactivty of Cs-137 (PBq)	Ambient dose by Cs-134 ($\times10^9$ Sv/h)	Ambient dose by Cs-137 ($\times10^9$ Sv/h)	Total ($\times10^9$ Sv/h)
2011/3/14	2018/7/18	2683	7.4	0.75	7.60	6.3	23.9	30.3
2011/3/14	2018/9/27	2754	7.5	0.70	7.57	5.9	23.8	29.8
2011/3/14	2018/10/13	2770	7.6	0.69	7.56	5.9	23.8	29.7
2011/3/14	2018/12/19	2837	7.8	0.65	7.53	5.5	23.7	29.2
2011/3/14	2019/1/12	2861	7.8	0.63	7.52	5.4	23.7	29.1
2011/3/14	2019/1/24	2873	7.9	0.63	7.51	5.3	23.7	29.0
2011/3/14	2019/2/27	2907	8.0	0.61	7.50	5.2	23.6	28.8
2011/3/14	2019/3/16	2924	8.0	0.60	7.49	5.1	23.6	28.7
2011/3/14	2019/4/25	2964	8.1	0.58	7.47	4.9	23.5	28.4
2011/3/14	2019/5/23	2992	8.2	0.56	7.46	4.8	23.5	28.3
2011/3/14	2019/7/4	3034	8.3	0.54	7.44	4.6	23.4	28.0
							physical decay =	92.5%

^{134}Cs (2.065 years of half-life) and ^{137}Cs (30.17 years of half-life) were equal 9.0, and 8.8 PBq at release time.

Ambient dose equivalent conversion coefficients for radionuclides exponentially distributed in the ground: Cs-134: 8.5 μSv/h per Bq/m^2; Cs-137: 3.15 μSv/h per Bq/m^2.[1]

1. Saito K, Petoussi-Henss N. 2014. Ambient dose equivalent conversion coefficients for radionuclides exponentially distributed in the ground. *Journal of Nuclear Science and Technology* 51:1274–1287. DOI: 10.1080/00223131.2014.919885.
